# Supplementary material for: Neutrophil Extracellular Traps Promote Platelet-Driven Contraction of Inflammatory Blood Clots via Local Generation of Endogenous Thrombin and Softening of the Fibrin Network
Source: Cells. 2025 Dec 18;14(24):2018. doi: 10.3390/cells14242018 (PMC12732175; doi:10.3390/cells14242018)
Supplement: Supplementary file 1 [file cells-14-02018-s001.zip › cells-4024150-supplementary.pdf]

SUPPLEMENTARY MATERIAL

Neutrophil Extracellular Traps Promote Platelet-Driven Contraction of Inflammatory Blood Clots via Local Generation of Endogenous Thrombin and Softening of the Fibrin Network

Short title: NETs promote contraction of inflammatory blood clots

Shakhnoza M. Saliakhutdinova[1,†], Rafael R. Khismatullin[1,†], Alina I. Khabirova[1], Rustem I. Litvinov[2], John W. Weisel[2]

[1]Department of Morphology and General Pathology, Institute of Fundamental Medicine and Biology, Kazan Federal University, Kazan, Russian Federation; [2]Department of Cell and Developmental Biology, University of Pennsylvania School of Medicine, Philadelphia, Pennsylvania, USA

†These authors contributed equally

Supplementary Figures

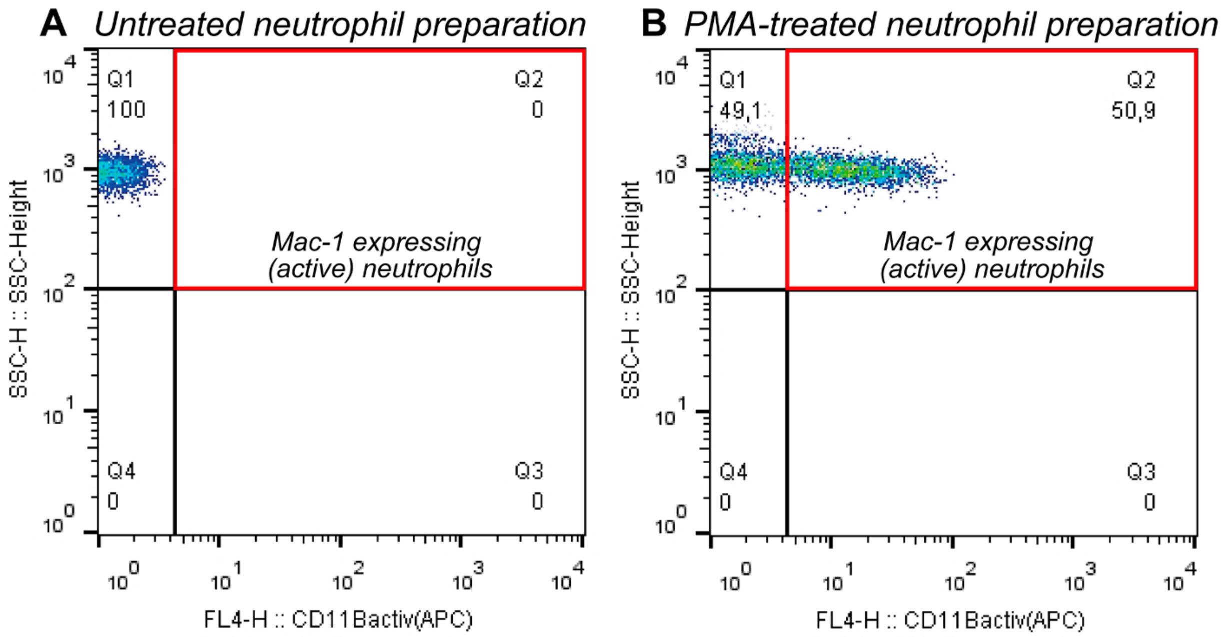

Figure S1. Representative flow cytometry of non-activated and phorbol-12-myristate-13-acetate (PMA)-activated isolated neutrophil preparations. No active Mac-1 (CD11b) expressed by neutrophils (Q2) in a control untreated neutrophil preparation (**A**) and a large fraction of CD11b+ neutrophils expressing active Mac-1 (Q2) in a PMA-treated neutrophil preparation (**B**), both determined within the gate of neutrophils with the absolute number of CD16+ signals taken as 100%.

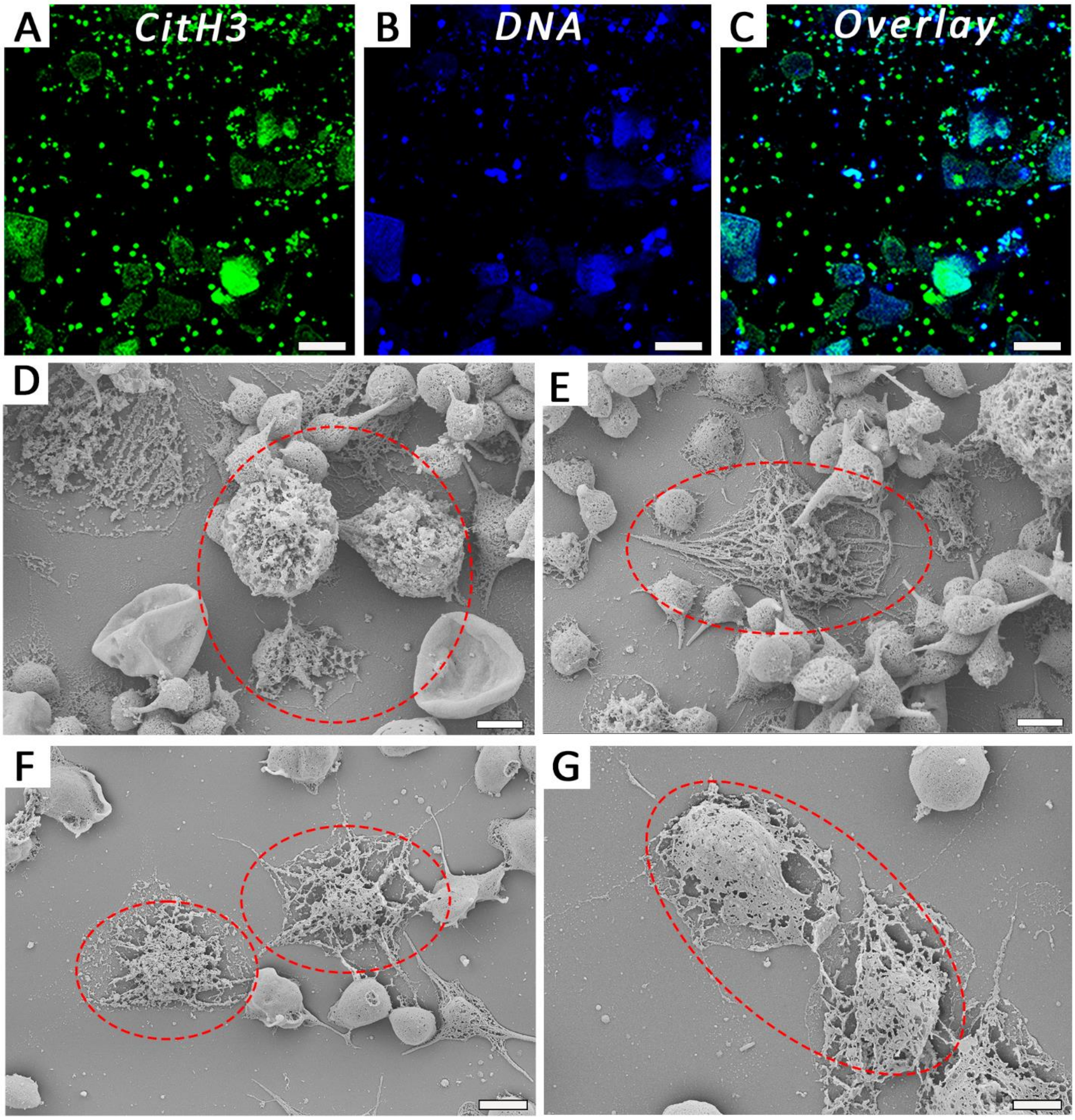

Figure S2. Neutrophil extracellular traps (NETs) formed by isolated PMA-activated neutrophils as visualized with immunohistochemistry and scanning electron microscopy. (A-C) Fluorescence micrographs showing PMA-treated neutrophil preparations stained for citrullinated histones CitH3 (*green*) and DNA (*blue*) to visualize neutrophils and NETs. CitH3 (A), deoxyribonucleic acid (DNA) (B), and overlay (C) images. **(D, E)** Representative high-resolution scanning electron micrographs of the ongoing NETosis (D) and a final isolated NET, not connected to a neutrophil (E). **(F. G)** NETs released by PMA-activated neutrophils into the supernatant, followed by precipitation on a coverslip (*inside dashed ovals*). NETs are surrounded by platelets and red blood cells. Magnification bars: 50 µm (A-C) and 2 µm (D-G).

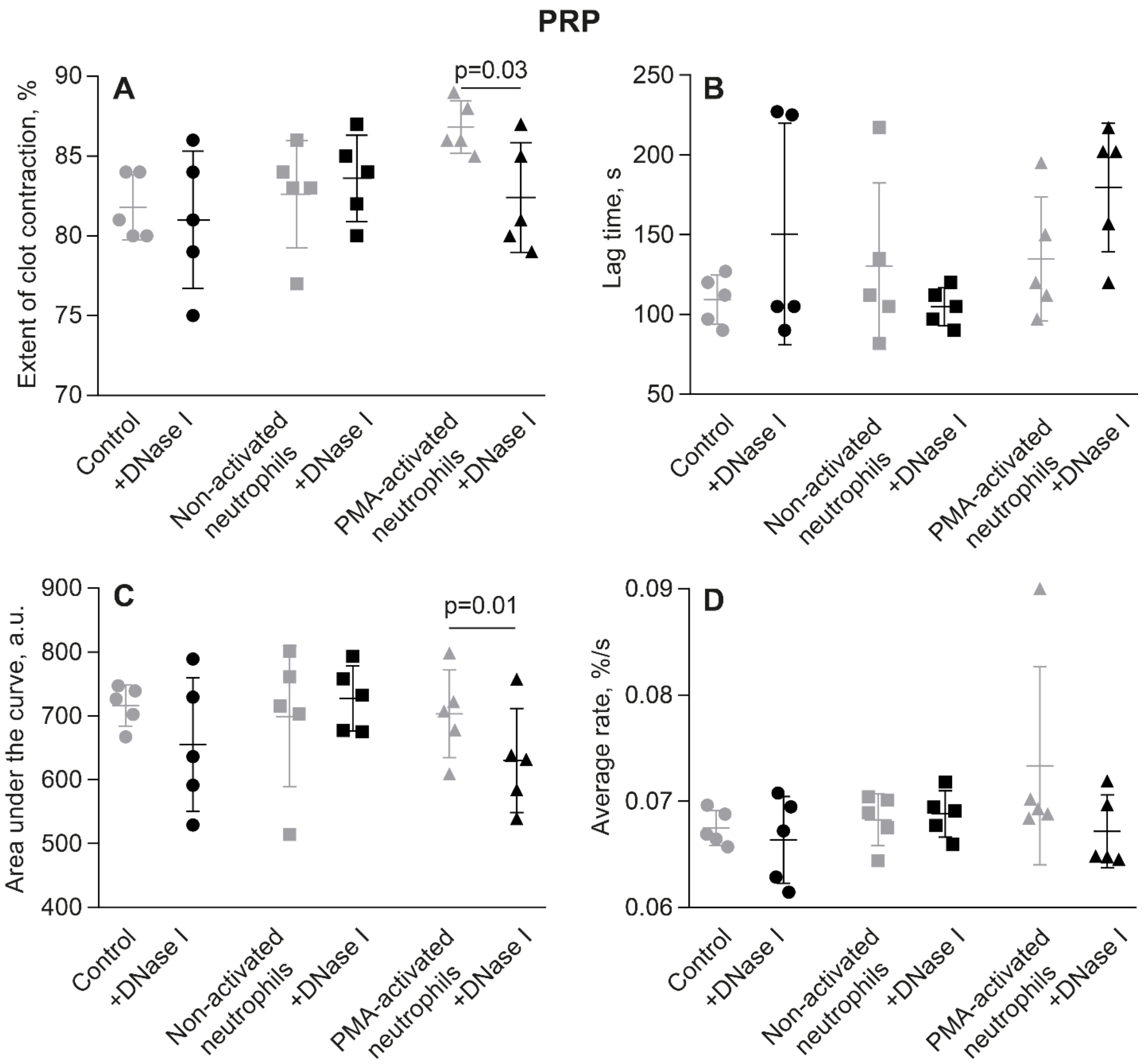

Figure S3. Deoxyribonuclease (DNase) I abrogates the promoting effects of PMA-activated neutrophils on clot contraction, but has no effect in the presence of non-activated neutrophils or by itself. The effects of DNase I on the parameters of clot contraction were studied in platelet-rich plasma (PRP) clotted in the absence (control) and presence of non-activated or PMA-activated neutrophils. Clot formation and contraction were induced by 1 U/ml thrombin followed by the optical tracking of a clot size. The final extent of clot contraction (**A**), lag time (**B**), area under the curve (**C**), and average rate (**D**) were measured in clots formed in PRP samples obtained from independent donors. Results are presented as the mean ± SD (n=5). The Student's paired *t*-test was used for comparisons. Only significant differences are shown. For numerical data and detailed statistical analysis see Table S5.

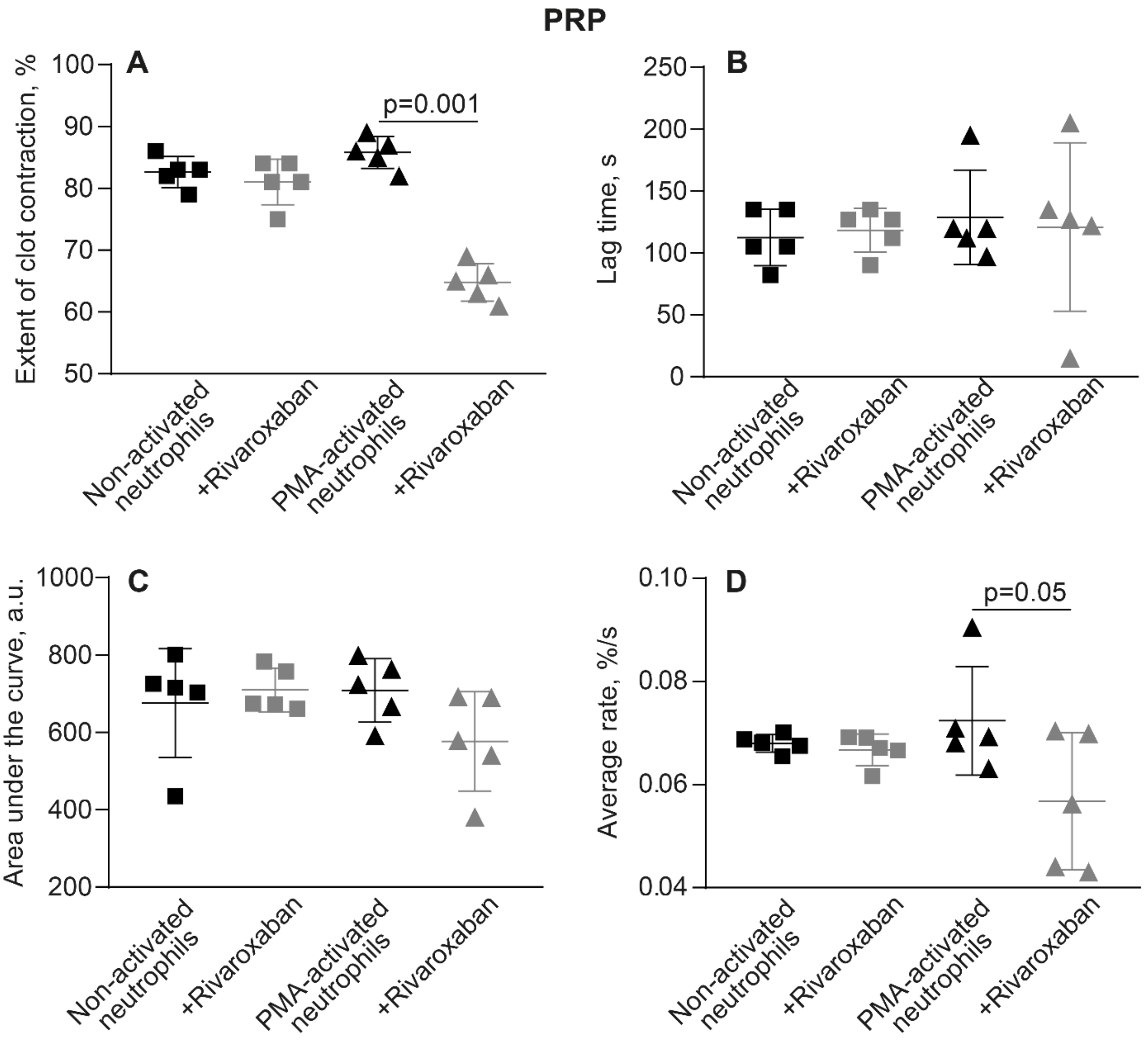

Figure S4. Rivaroxaban abrogates the promoting effects of PMA-activated neutrophils on PRP clot contraction, but has no effect in the presence of non-activated neutrophils. Clot formation and contraction were induced by 1 U/ml thrombin followed by the optical tracking of the clot size. The final extent of clot contraction (**A**), lag time (**B**), area under the curve (**C**), and average rate (**D**) were measured in clots formed in PRP samples obtained from independent donors. Results are presented as the mean ± SD (n=5). The Student's paired *t*-test was used for comparisons. Only significant differences are shown. For numerical data and detailed statistical analysis see Table S7.

Supplementary Tables

Table S1. Parameters of clot contraction in whole blood and platelet-rich plasma (PRP) clotted in the absence and presence of non-activated or phorbol-12-myristate-13-acetate (PMA)-activated neutrophils

Parameters of clot contraction	*Control (no added neutrophils)*	*Non-activated neutrophils*	*PMA-activated neutrophils*	*p-values*
**Whole blood**				
Final extent of clot contraction (%)	44±3	45±3	50±5	***0.046***
Lag time (sec)	147±30	63±48	39±16	***0.001***
Area under the kinetic curve (a.u.)	351±42	426±72	427±86	0.2
Average rate (%/sec)	0.036±0.002	0.036±0.002	0.039±0.003	0.07
**PRP**				
Final extent of clot contraction (%)	80±1	80±1	86±3	***0.0005***
Lag time (sec)	99±4	94±10	108±21	0.4
Area under the kinetic curve (a.u.)	706±27	743±25	718±34	0.2
Average rate (%/sec)	0.066±0.001	0.065±0.001	0.070±0.003	***0.002***

Results are presented as the mean ± SD (n=5 for whole blood; n=4 for PRP using the blood of independent donors). One-way ANOVA.

Table S2. Parameters of clot contraction in PRP in the absence (control) and presence of 22 nM PMA, corresponding to the final concentration of PMA added with PMA-activated neutrophil preparations

Parameters of clot contraction	*PRP (control)*	*PRP + 22 nM PMA*	*p-values*
Final extent of clot contraction (%)	81±2	82±1	0.06
Lag time (sec)	96±14	118±13	0.17
Area under the kinetic curve (a.u.)	729±30	698±17	0.63
Average rate (%/sec)	0.066±0.001	0.067±0.001	0.43

Results are presented as the mean ± SD (n=4). The Student's paired *t*-test.

Table S3. Parameters of comparative phase analysis (phase duration and rate constant) of the averaged kinetic curves of contraction of clots formed in the whole blood and PRP

Phases	*Non-activated neutrophils*	*PMA-activated neutrophils*	*p-values*	*Non-activated neutrophils*	*PMA-activated neutrophils*	*p-values*
	Duration, sec			Rate constant, 1/s		
**Whole blood**						
I	45±39	98±34	***0.02***	0.04±0.06	0.02±0.01	0.8
II	132±47	129±70	0.9	0.10±0.02	0.07±0.02	***0.002***
III	1023±32	999±88	0.4	0.005±0.001	0.003±0.001	***0.0002***
**PRP**						
I	23±11	47±8	***0.02***	Immeasurable	0.39±0.33	-
II	105±8	126±40	0.2	0.16±0.03	0.15±0.06	0.6
III	1073±8	1028±42	***0.03***	0.005±0.001	0.004±0.001	***0.004***

Results are presented as the mean ± SD (n=5 for whole blood; n=4 for PRP using the blood of independent donors). For whole blood clots, the Wilcoxon (non-parametric) test for phase 1 and the Student's paired (parametric) *t*-test for phases 2 and 3 were used. For PRP clots, the Wilcoxon (non-parametric) test for durations and the Student's paired (parametric) *t*-test for rate constants were used.

Table S4. Effects of deoxyribonuclease (DNase) I on the parameters of clot contraction in whole blood clotted in the absence (control) and presence of non-activated or PMA-activated neutrophils

Parameters of clot contraction	*Control (no added neutrophils)*	*+ DNase I*	*Non-activated neutrophils*	*+ DNase I*	*PMA-activated neutrophils*	*+ DNase I*
Final extent of clot contraction (%)	46±1	47±4	47±2	46±6	54±2	44±5
p-values	0.4		0.5		***0.02***	
Lag time (sec)	60±31	48±22	55±25	100±52	28±12	36±13
p-values	0.6		***0.04***		0.2	
Area under the kinetic curve (a.u.)	382±26	395±73	374±65	363±80	438±44	354±56
p-values	0.8		0.8		0.07	
Average rate (%/sec)	0.036± 0.002	0.038± 0.002	0.036± 0.005	0.037± 0.004	0.041± 0.002	0.035± 0.004
p-values	0.5		0.8		***0.04***	

Results are presented as the mean ± SD (n=5). The Student's paired *t*-test.

Table S5. Effects of DNase I on the parameters of clot contraction in PRP clotted in the absence (control) and presence of non-activated or PMA-activated neutrophils

Parameters of clot contraction	*Control (no added neutrophils)*	*+ DNase I*	*Non-activated neutrophils*	*+ DNase I*	*PMA-activated neutrophils*	*+ DNase I*
Final extent of clot contraction (%)	82±2	81±4	83±3	84±3	87±2	82±3
p-values	0.7		0.6		***0.03***	
Lag time (sec)	109±15	150±69	130±52	105±12	135±39	180±40
p-values	0.3		0.5		0.1	
Area under the kinetic curve (a.u.)	716±32	655±105	699±110	727±51	703±69	630±82
p-values	0.2		0.6		***0.01***	
Average rate (%/sec)	0.067± 0.002	0.066± 0.004	0.068± 0.002	0.069± 0.002	0.073± 0.009	0.067± 0.003
p-values	0.6		0.7		0.09	

Results are presented as the mean ± SD (n=5). The Student's paired *t*-test.

Table S6. Effects of rivaroxaban on the parameters of clot contraction in whole blood clotted in the absence and presence of non-activated or PMA-activated neutrophils

Parameters of clot contraction	*Non-activated neutrophils*	+ *Rivaroxaban*	*PMA-activated neutrophils*	+ *Rivaroxaban*
Final extent of clot contraction (%)	48±5	42±5	55±3	39±4
p-values	0.08		***0.003***	
Lag time (sec)	93±40	76±29	34±18	43±15
p-values	0.6		0.3	
Area under the kinetic curve (a.u.)	388±48	347±37	425±41	312±45
p-values	0.2		***0.02***	
Average rate (%/sec)	0.039± 0.003	0.034± 0.003	0.039± 0.003	0.032± 0.003
p-values	0.07		***0.03***	

Results are presented as the mean ± SD (n=5). The Student's paired *t*-test.

Table S7. Effects of rivaroxaban on the parameters of clot contraction in PRP clotted in the presence of non-activated or PMA-activated neutrophils

Parameters of clot contraction	*Non-activated neutrophils*	*+ Rivaroxaban*	*PMA-activated neutrophils*	*+ Rivaroxaban*
Final extent of clot contraction (%)	83±3	81±4	86±3	65±3
p-values	0.4		***0.001***	
Lag time (sec)	112±23	118±18	129±38	121±68
p-values	0.6		0.5	
Area under the kinetic curve (a.u.)	676±140	709±56	709±82	577±128
p-values	0.7		0.1	
Average rate (%/sec)	0.068±0.002	0.067±0.003	0.072±0.010	0.057±0.013
p-values	0.4		0.053	

Results are presented as the mean ± SD (n=5). The Student's paired *t*-test.

Table S8. Parameters of thromboelasography (TEG) - the reaction time (*R*) and clot elastic modulus (*G′*) - in the absence (control) presence of non-activated and PMA-activated neutrophils

Parameters of TEG	*Control (no added neutrophils)*	*Non-activated neutrophils*	*PMA-activated neutrophils*	*p-values*
**Whole blood**				
R (min)	2.1±0.5	1.9±0.6	2.3±0.7	0.7
G′ (Pa)	341±119	498±220	336±173	0.3
**PRP**				
R (min)	1.2±0.3	1.0±0.2	1.2±0.1	0.3
G′ (Pa)	931±190	1225±236	807±274	***0.04***

Results are presented as the mean ± SD (n=5). One-way ANOVA.

Table S9. Effect of DNAse I on the parameters of thromboelastogram (TEG) in the absence (control) and presence of non-activated or PMA-activated neutrophils in whole blood and in PRP

Parameters of clot contraction	*Control (no added neutrophils)*	*+ DNase I*	*Non-activated neutrophils*	*+ DNase I*	*PMA-activated neutrophils*	*+ DNase I*
**Whole blood**						
Reaction time, *R* (min)	2.1±0.5	1.5±0.8	1.9±0.6	1.5±0.4	2.3±0.7	1.6±0.4
p-values	0.1		0.1		***0.03***	
Clot elasticity, *G′* (Pa)	341±119	547±231	498±220	633±327	336±173	481±180
p-values	0.3		0.2		***0.01***	
**PRP**						
R (min)	1.2±0.3	0.9±0.1	1.0±0.2	0.9±0.2	1.2±0.1	1.1±0.2
p-values	0.1		0.7		0.2	
G′ (Pa)	931±190	868±271	1225±236	916±489	807±274	977±243
p-values	0.5		0.2		0.4	

Results are presented as the mean ± SD (n=5). The Student’s paired *t*-test.
